# Correlation of HbA1c with Serum Iron & Transferrin Saturation in Non-Diabetic Patients with Iron Deficiency

**DOI:** 10.12669/pjms.39.4.6964

**Published:** 2023

**Authors:** Darshan Kumar, Tazeen Rasheed, Bader Faiyaz Zuberi, Rabiah Sadaf, Faiza Sadaqat Ali

**Affiliations:** 1 Darshan Kumar (FCPS) Professor, Department of Medicine DIKIOHS, Dr. Ishrat-ul-Abad Institute of Oral Health Sciences, Dow University Health Sciences Karachi; 2 Dr. Tazeen Rasheed (FCPS) Associate Professor, Department of Medicine, Dow Medical College, Dow University of Health Sciences, Karachi, Pakistan; 3 Bader Faiyaz Zuberi (FCPS) Meritorious Professor, South City Hospital, Karachi, Pakistan; 4 Dr. Rabiah Sadaf (FCPS) Consultant Physician, Ruth KM Pfau Civil Hospital, Karachi, Pakistan; 5 Faiza Sadaqat Ali (FCPS) Senior Registrar, Department of Medicine, Dow Medical College, Dow University of Health Sciences, Karachi, Pakistan

**Keywords:** HbA1c, Iron deficiency, Serum iron, Transferring saturation, Non-diabetic

## Abstract

**Objective:**

To determine correlation of HbA1c with serum iron and transferrin saturation in non-diabetic patients with iron deficiency.

**Methods:**

This cross-sectional comparative study was conducted at Dr Ruth KM Pfau Civil Hospital Karachi from 15^th^ September 2021 to 14^th^ March 2022, on non-diabetic patients based on fasting blood sugar (FBS) of <100 mg/dl. Patients were divided into two groups. Group one included patients having iron deficiency anemia (ID), whereas group two included same number of age and sex matched healthy subjects taken as controls, without ID. Blood sample was taken for HbA1c, CBC, serum iron and total iron binding capacity. Transferrin saturation (TSAT) was calculated. Comparison of quantitative variables with ID and non-ID group was done by Student’s t-test. Correlation of HbA1c with iron &TSAT was done in both groups using Kendal tau-b test, as data was not normally distributed.

**Results:**

Out of 230 patients, 83 (36.1%) were males while 147(63.9%) were females. Mean age of patients was 43.7±13.28 years. Mean HbA1c level was significantly high in ID group (5.89±0.43) as compared to non-ID group (5.52±0.50) with a p-value <.001. The HbA1c levels correlated negatively with hemoglobin, serum iron levels and transferrin saturation with a p-value <.001.

**Conclusion:**

Low serum Iron and TSAT was related to elevation in HbA1c value. Iron deficiency needs to be corrected before HbA1c interpretation.

## INTRODUCTION

Hemoglobin A1C (HbA1c) is the key form of glycosylated hemoglobin.^1^ It is formed by ketamine reaction between N-terminal amino acid of the β-chain of hemoglobin and serum glucose. Fraction of glycosylated hemoglobin is proportional to the average plasma glucose levels. HbA1c in diabetic patients reflects the plasma glucose levels of the past three months.^2^ “The American Diabetes Association (ADA) recommends using HbA1c to diagnose diabetes”.^3^ Previously it was suggested that HbA1c is only affected by plasma glucose levels,^4^ but recent studies have shown that there are a lot of factors other than diabetes which affect its levels like hemoglobinopathies, hemolytic anemia’s, chronic kidney disease, alcoholism, pregnancy and dietary anemias.^5^,^6^

Iron deficiency (ID) is the most common type of nutritional deficiency. ID is a major public health problem that is prevalent worldwide and has serious adverse effects on human health in addition to its negative impact on economic development.^7^ Overall, ID is responsible for 50% of all the anemias. Studies have shown that decrease in iron level leads to increase glycation of HbA1c.The other theories about increase level of HbA1c in ID are:

- More rapid glycation of globin chain due to change in the quaternary structure.^8^
- In iron deficiency anemia, there is a decrease in red blood cells production that increases the life span of red cells and thus increase the HbA1c level.
- Due to decrease in the hemoglobin level there is an increase in the glycation of hemoglobin resulting increase in HbA1c levels.^9^,^10^

Since the glycemic control of an individual is reflected by the HbA1c levels, therefore it is important to exclude factors which could erroneously elevate its levels.^11^ Previous studies have suggested that iron-deficiency anemia influences HbA1c levels, but the results were contradictory.^9^-^13^ Son NE et al. used serum ferritin levels as marker for iron deficiency to correlate with HbA1c.^14^ Similarly Son NE reported increased serum ferritin levels with increasing HbA1c levels in patients with type 2 DM,^14^ but as ferritin is an acute phase reactant, its results could be invalid. We did not find any local study which studied correlation of serum iron and transferrin saturation, which are more reliable markers for iron deficiency with HbA1c.

Since iron deficiency anemia is very common in Pakistan, the prevalence is reported to be around 45% according to a study by Mawani et al,^15^ therefore, we conducted this study to assess the effect of ID on HbA1c levels in non-diabetic patients, and to determine if ID should be considered before making any therapeutic decisions based solely on HbA1c levels.

## METHODS

This cross-sectional comparative study was conducted at Dr. Ruth KM Pfau Civil Hospital Karachi between 15^th^ September 2021 to 14^th^ March 2022. Approval (IRB-2141/DUHS/Approval/2021/ Dated 10^th^ September 2021) was taken from “Institutional Review Board of Dow University of Health Sciences”. Informed written consent was taken from all patients. All non-diabetic patients between the ages of 17 to 70 years were included and were segregated into two groups based on serum iron and transferrin saturation. Patients who were not known diabetics and were having fasting blood sugar (FBS) levels <100mg/dl were considered non-diabetics and patients whose serum iron was <59 their HbA1c parameter usedμg/dl^16^ and Serum Transferrin saturation <15%^17^ were labelled as Iron Deficient.

Patients with serum iron of <59 μg/dl and transferrin saturation of <15% were allocated to Group-1 and those with serum iron of ≥59 μg/dl and transferrin saturation of ≥15% were allocated to Group-2. Patients with a history of acute blood loss, hemolytic anemia, hemoglobinopathies, chronic kidney disease, alcohol ingestion, vitamin B_12_ and folate deficiencies were excluded. Patients taking drugs which can affect the HbA1c levels like corticosteroids, anti-retroviral, ribavirin etc. were also excluded. Calculation of sample size was done by PASS 2019 software. Sample size was calculated using power of 0.90 to detect a difference of -0.26 between the null hypothesis correlation of <0.001 and the alternative hypothesis correlation of 0.26 using a two-sided hypothesis test with a significance level of 0.05.^18^ Using the parameters described sample size was calculated as 152.

### Data Collection:

Non-diabetic patients fulfilling selection criteria were inducted after informed written consent. Demographic details were recorded. 10 ml of venous sample was taken for HbA1c, complete blood count, serum iron and total iron binding capacity (TIBC). Transferrin saturation was calculated by the formula. Transferrin saturation (%) = serum iron level (μg/dL) / TIBC (μg/dL) x 100. Serum iron and HbA1c were measured by HPLC method using Bio-Rad D10 analyzer. Patients were instructed to report in fasting state for eight hours for plasma glucose which was estimated by oxidase/peroxidase method. Patients with iron deficiency as per operational definition were allocated to Group-1 and those who were not iron deficient were allocated to Group-2.

### Data Analysis:

Frequencies of qualitative variables like gender and ID status were determined and compared by x^2^ test. Means ±SD of quantitative variables like age, HbA1c, iron, transferrin saturation was determined. Comparison of quantitative variables of age, HbA1c, iron & transferrin saturation with gender and ID Groups was done using t-test. Normal distribution of all quantitative variables was checked by Kolmogorov-Smirnov (KS) Test. KS Test value was <0.05 showing that the data was not normally distributed. Thus, correlation of HbA1c with iron and transferrin saturation was done using with Kendall’s Tau-b test. Significant level was set at ≤0.05. SPSS version 26.0 was used for data analysis.

## RESULTS

A total of 230 patients were inducted in the study after informed consent. Mean age of the patients was 43.7 ± 13.28 years. There were 147(63.9%) females and 83 (36.1%) males. Mean age of females was 42.9 ± 12.38 years while that of males was 45.3 ± 14.7 years. There was no significant difference in age & HbA1c between genders. Significant difference was found between gender among hemoglobin, serum iron, and transferrin saturation, details are shown in Table-I.

**Table-I:** Comparison of quantitative variables with gender by Student’s t-test.

	Gender	Mean ±SD	Sig.	95% CI for Mean	

				Upper	Lower
Age (years)	Female	42.90 ±12.38	.185	1.167	-6.008
	Male	45.33 ±14.70			
Haemoglobin (gm/dl)	Female	9.90 ±1.99	.042*	0.458	0.669
	Male	11.00 ±2.25			
HbA1c	Female	5.72 ±0.53	.357	0.071	-0.197
	Male	5.79 ±0.44			
Serum Iron (μg/dl)	Female	45.40 ±22.83	.037*	5.939	7.007
	Male	50.94 ±25.77			
Transferrin Saturation (%)	Female	29.12 ±9.87	.039*	29.043	12.102
	Male	35.44 ±12.23			

* . Significant level ≤.05.

There was no significant difference between Group-1 and Group-2 with regards to age; t (228) = -1.916, p = 0.057. However, significant difference was found with regard to Hb, serum iron and HbA1c levels. Mean HbA1c level was significantly high in Group-1 (5.89 ± 0.43) as compared to Group-2 group (5.52 ± 0.50) with a p vale of <.001. Details of which are given in Table-II.

**Table-II:** Comparison of age, HbA1c, serum iron & transferrin saturation with ID Groups by Student’s t-test.

	Groups	Mean ±SD	Sig.	“95% Confidence Interval for Mean”	

				Upper	Lower
Age (years)	Group-1	45.15 ±13.17	.057	0.103	-6.904
	Group-2	41.75 ±13.25			
HbA1c	Group-1	5.89 ±0.43	<.001*	-0.253	-0.498
	Group-2	5.52 ±0.50			
Serum Iron (μg/dl)	Group-1	33.65 ±13.30	<.001*	45.09	38.69
	Group-2	75.55 ±10.02			
Transferrin Saturation (%)	Group-1	8.94 ±3.42	<.001*	23.04	12.78
	Group-2	39.64 ±7.12			

* . Significant level ≤.05.

As data was not normally distributed, correlation of hemoglobin with HbA1c, serum Iron was studied by non-parametric test of Kendall’s tau-b. The HbA1c levels correlated negatively with hemoglobin, serum iron levels and transferrin saturation. Details of which are given in Table-III.

**Table-III:** Correlation matrix of HbA1c with Hemoglobin, Serum Iron and Transferrin Saturation.

		Hemoglobin (gm/dl)	Serum Iron (mcg/dl)	Transferrin Saturation (%)
HbA1c	Correlation Coefficient	-.286**	-.288**	-.291**
	Sig. (2-tailed)	<.001*	<.001*	<.001*

* . Significant level ≤.05,

** . “Correlation is significant at the .01 level (2-tailed)”.

## DISCUSSION

Two major findings were reported in our study, first that patients with iron deficiency have higher HbA1c levels as compared to those who don’t and secondly the levels of HbA1c have a strong negative correlation with hemoglobin, serum iron and transferrin saturations.

Iron deficiency is a common prevalent nutritional deficiency that is commonly encountered in clinical practice accounting 50% of all anemias globally.^19^ Iron deficiency is linked to increased HbA1c levels.^13^ HbA1c divulges glycemic status for previous three months and is also used to diagnose diabetes and pre-diabetes, i.e., patients at high risk for developing diabetes.^20^ Factors, such as ethnicity, age, genetics and certain diseases can effect HbA1c values.^21^ Previous studies have shown that ID may upraise HbA1c level, independent of glycemic level.^11^ Therefore, HbA1c measure may not precisely represent glycemic status in patients having certain anemias, blood loss or ID. Intra J et al, in his study also concluded that, in anemic individuals, the adjusted means of HbA1c were notably raised (5.59% 37.37 mmol/mol), as compared to individuals without anemia (5.34% 34.81 mmol/mol) (P<0.001).^12^ Previous studies have also shown that in individuals with or without diabetes, ID is associated with elevated HbA1c values, which decrease upon correction of ID.^12^,^22^

In this study we found statistically significant difference between the ID and non-ID groups with regards to HbA1c levels, with the higher levels of HbA1c in the ID group. Madhu et al.^22^ reported significantly higher HbA1c levels in ID subjects (5.51 ± 0.696) compared to healthy control group (4.85 ± 0.461%); p <.001. They observed negative correlation between HbA1c and hemoglobin, hematocrit, RBC count, MCH, MCHC and serum ferritin in ID subjects (r=-0.632, -0.652, -0.384, -0.236, -0.192 and -0.441) and after iron replacement, remarkable decline was seen in HbA1c levels in ID subjects (5.51+/-0.696 before treatment v/s 5.044 ±0.603 post-treatment; p<.001). Bansal et al.^23^ also observed statistically significant difference (p value <.001) between ID and control group with mean HbA1c of 6.11 ± 0.42 and 5.01 ± 0.41 respectively. Cetinkaya Altuntas S et al. in their study showed that IDA was associated with low HbA1c levels, and increased after iron therapy.^1^ However, Coban E et al.^24^ reported increased HbA1c levels in patients with IDA. They supplemented the patients of ID with iron 100mg/day for three months and followed the HbA1c of the patients thereafter. They found that the HbA1c levels decreased significantly to 6.2% ±0.6 (p <.001) after iron therapy.

Similar results were presented by Bukhari et al.^25^, Silva et al.^26^ and Christy et al.^11^ However, Sinha et el.^13^ reported contradictory results with HbA1c level in patients of ID lower than those in the control group (4.6% in ID group versus 5.5% in the control group, P<.05). They also reported in their study a significant increase in HbA1c levels after two months of iron therapy (0.29 g/dL vs. 0.73 g/dl, p <.01). Although the exact reason for the increase in HbA1c levels in ID are not known but few of the studies reported this.

### Limitations:

Small sample size and lack of follow up after iron therapy are few limitations of the study. This study was done in non-diabetic patients and it is possible that the effect of anemia on HbA1c levels may be greater, which could have significant clinical relevance in patients with DM. Due to hospital-based design and single center, the results of this study could not be generalized to general population.

## CONCLUSION

This study statistically proved the correlation of ID and HbA1c levels, Thus, diabetic population should periodically check their iron status too, that has the potency to influence their HbA1c parameter used to assess the degree of their glycaemic control before.

### Authors’ Contribution:

**DK, TR and BFZ:** Substantial contributions to conception, design, acquisition of data, analysis and interpretation of data.

**RS and FSA:** Drafting the article or revising it critically for important intellectual content.

**BFZ:** Final approval of the version to be published.

**TR and FSA:** Statistical Analysis.

All Authors agreed to be accountable for all aspects of the work in ensuring that questions related to the accuracy or integrity of any part of the work are appropriately investigated and resolved.
